# Perceived stress, coping mechanisms, and influential factors among undergraduate nursing students during ICU clinical placements: A cross-sectional study

**DOI:** 10.1371/journal.pone.0323406

**Published:** 2025-05-09

**Authors:** Osama Alkouri, Ahmad M. Al-Bashaireh, Yousef Aljawarneh, Zainab Albikawi, Mohammad Abuadas, Alanoud Alobaidly, Omar Qaladi, Abdulhafith Alharbi, Abdulkareem Alshehri, Ghaya Alblooshi, Taif Alsayegh, Fatmah Alkaabi, Shahad Alyammahi, Hanadi Alhafety, Ahmad Rajeh Saifan

**Affiliations:** 1 Department of Basic Nursing, Yarmouk University, Irbid, Jordan; 2 Department of Nursing, Faculty of Health Sciences, Higher Colleges of Technology, Abu Dhabi, United Arab Emirates; 3 Department of Clinical Nursing, Yarmouk University, Irbid, Jordan; 4 Nursing and Health Sciences, Flinders University, Adelaide, Australia; 5 Community and Psychiatric Mental Health, King Saud University, Riyadh, Saudi Arabia; 6 Psychiatric and Mental Health Nursing, University of Hail, Hail, Saudi Arabia; 7 Advanced Diagnostic and Therapeutic Institute, King Abdulaziz City for Science and Technology (KACST), Riyadh, Saudi Arabia; University of Sharjah, UNITED ARAB EMIRATES

## Abstract

**Background:**

Intensive Care Unit (ICU) exposes nursing students to high workloads, emotional demands, and high-risk performance. Understanding perceived stress, coping strategies, and influential factors may enhance students’ clinical experiences and outcomes.

**Aim:**

To assess perceived stress levels, identify coping mechanisms, and explore associations between stress, coping mechanisms, and demographic factors among nursing students during their Intensive Care Unit (ICU) clinical.

**Setting:**

The study was conducted across three campuses of the Higher Colleges of Technology (HCT) in the United Arab Emirates (UAE).

**Methods:**

A cross-sectional study design with a total sample of 127 undergraduate nursing students was conducted. Data were collected using the Perceived Stress Scale (PSS) and Coping Behavior Inventory (CBI).

**Results:**

Students reported a moderate level of perceived stress (mean = 1.87, SD = 0.80). The highest-ranking stressors reported included assignments and workload, with (mean = 2.12, SD = 0.91), followed by peer-related stress (mean = 1.98, SD = 1.03). The most reported coping mechanism among students was the problem-solving mechanism (mean = 2.23, SD = 0.95), followed by the transference mechanism (mean = 2.17, SD = 1.00), and staying optimistic (mean = 2.15, SD = 0.95). Stepwise regression showed that the significant predictors of overall stress were avoidance coping, β = 0.65, p < 0.001, and transference coping, β = 0.24, p < 0.001, explaining 63% of variance, R² = 0.63. Problem-focused coping negatively predicted environmental stress, (β = -0.21, p = 0.021), highlighting its protective role.

**Conclusion:**

This research underscores the nursing students’ moderate stress experienced during ICU nursing placements due to workload and peer pressure. Use of problem-focused coping strategies reduced stress, while maladaptive avoidance coping strategies increased stress. Stress was strongly predicted by avoidance and transference coping confirming the necessity for coping skills instruction in nursing. Teaching stress coping and resilience building in clinical education will improve students’ well-being, performance, and preparedness for critical care nursing.

## Introduction

Nursing education prepares students both theoretically and practically for future practice. It provides clinical placements within different healthcare settings, including hospitals and specialist units. Clinical placements are structured to enhance nursing students’ competence and confidence in managing complex patient scenarios [1]. Clinical learning in high-intensity environments such as the Intensive Care Unit (ICU) offers unrivaled learning opportunities amidst a host of challenges. These are mainly because of the stressful and high-stakes nature of such settings [2].

Research has implicitly supported the fact that during clinical placements, the students are exposed to a number of stressors which emanate from patient care demands, intricacy of clinical tasks, and the emotional load related to managing critically ill patients [3]. Furthermore, research indicates that nursing students are usually exposed to high perceived levels of stress in clinical placements, especially in ICUs. Mean stress scores ranging from moderate to high among nursing students have been reported by various studies. These provoke the emergence of specific stressors related to the clinical environment, workload, and emotional demands of the patients [4].A systematic review showed that even the shift from theoretical learning to practical application in high-pressure settings, such as ICUs, heightens the level of stress among students grappling with the complexities of the practice entailed in caring for patients and fear of committing mistakes [5].

The stressors experienced by nursing students in ICUs are multifaceted. High workload, emotional demands of caring for their patients, and pressures to meet expectations of educators and clinical staff have been identified as major ones [4,6]. Specific factors identified include fear of approaching dissolution, feelings of incompetence and discrepancy between theoretical and practical knowledge. These were recorded as very important sources of stress in many studies [6,7]. These factors are further compounded by factors of interpersonal relationships occurring between themselves and clinical staff and the novelty of the ICU environment [7].In addition, clinical placement exposes nursing students to various types of stressors, which include pressure related to patient care, and the complexity of clinical skills to be executed, along with a psychological burden constituting the management of critically ill patients [3,8]. Consequently, the outcome of these factors is feeling incompetent and anxious, especially among those who have little experience in the ICU setting [9,10].

Nursing students employ a variety of coping strategies to manage stress during their ICU placements. Problem-solving approaches have been reported rather constantly as the most helpful ones for letting students go directly to and confront their specific difficulties [11]. Social support, physical activities, and relaxation techniques are also commonly used [12]. However, some students quickly turn to maladaptive coping strategies, such as denial or substance use, which may reflect unfavorably on their overall well-being [13]. Research, for example, has revealed that about 10% of nurses are chemically dependent and that for many of them, substance misuse starts while they are in nursing school [14]. A multicenter study including nursing schools from Spain, Belgium, France, and Brazil also revealed significant levels of illegal drug use among nursing students, hence stressing elements including nationality and gender that may affect use [15]. Moreover, coping skills are generally effective in relation to the level of resilience and self-efficacy of the students [7,16].

Variations in perceived stress levels and coping mechanisms among nursing students can be influenced by demographic factors such as age, gender, and year of study. For instance, it is found that across many studies, female nursing students tend to report higher levels of stress compared to male nursing students, which could be linked to a difference in their coping styles and social expectations [17]. Besides, students in earlier years of their education are likely to experience heightened levels of stress due to a lack of experience and confidence in clinical settings [18,19]. The relationship between perceived stress, coping mechanisms, and demographic variables is complex. Research has documented that higher levels of perceived stress are generally accompanied by less effective coping strategies, which can be further exacerbated by lower levels of social support and self-esteem [20]. Demographic variables such as age and education level often influence perceived stress and choice of coping strategies; thus, supportive systems in nursing education need to be specific and relevant within the context of the individuals concerned [18,20].

The significance of addressing stress and coping mechanisms among nursing students in the United Arab Emirates (UAE) is underlined by the very demands and peculiarities of stressors associated with clinical traineeship, especially in the ICU settings. The challenges of critical care, high mortality rates in an ICU environment, and the demands for swift clinical decision-making raise perceived stress levels and markedly affect learning and professional growth among the UAE’s nursing students [21]. There is an increasing need for evidence-based strategies that would support students’ mental well-being and effective coping, as such factors impact retention, competency, and resilience in future healthcare professionals. Furthermore, demographics and individual differences affecting stress and coping must be taken into consideration when providing support. Attention to such aspects is likely to go a long way in improving the clinical learning experience of the student and preparing the budding nurses for the various challenges that are an inherent component of critical care nursing. Therefore, the current study aimed to assess perceived stress levels, identify coping mechanisms, and explore associations between stress, coping mechanisms, and demographic factors among nursing students during their Intensive Care Unit (ICU) clinical placements. Moreover, this study specifically explored the following questions:

1. What are the levels of perceived stress among nursing students during their Intensive care Unit (ICU) clinical placements?
2. What are nursing students’ most experienced stressors during their Intensive care Unit (ICU) clinical placements?
3. What are nursing students’ most common coping mechanisms during their Intensive care Unit (ICU) clinical placements?
4. Are there any differences in the average scores of perceived stress and coping mechanisms across nursing students’ demographics during their Intensive care Unit (ICU) clinical placements?
5. Is there any relationship between perceived stress and coping mechanisms and demographics among nursing students during their Intensive care Unit (ICU) clinical placements?

## Research methods and design

### Research design

This study deployed a cross-sectional design to assess perceived stress levels and identify nursing students’ coping mechanisms during their Intensive care Unit (ICU) clinical placement.

### Population, sample, sampling procedure and sample size estimation

This study focused on fourth year undergraduate nursing students enrolled in Higher College of Technology (HCT). Three campuses, Sharjah, Ras Al-Khaimah, and Fujairah—offer the nursing program, with a total of 612 nursing students. The distribution across campuses is as follows: Ras Al-Khaimah has 124 students, Sharjah has 203, and Fujairah has 285. Moreover, the population in this study will be only students who currently undergo Intensive Care Unit (ICU) clinical rotation. Students in the fourth year who were in semester 7 and 8 and able to read and understand English was the targeted population. The statistics of HCT show the targeted population number is 180, and they were distrusted as follows: 86 in Fujairah Campus (42 level 7, 46 level 8), 60 in Sharjah campus (40 level 7, 20 level 8), and 34 in Ras Al-Khaimah campus (20 level 7, 12 level 8).

Convenience sampling was utilized to draw the sample for this study, a non-probability sampling method where a sample is drawn from an existing population. It is a pragmatic approach for researchers, quick, cost-effective, efficient, and straightforward to implement.

The sample size was calculated based on representativeness rather than statistical adequacy and it’s fair to answer questions related to the primary aim of this study. The online Raosoft’s sample size formula was used in this study. A minimum of 123 participants is required, given that the margin of error alpha (α) = 0.05, the confidence level is = 95%, total population = 180, and the response of distribution = 50%. However, based on a small sample frame and to answer questions under secondary aims, the whole 180 subjects were targeted. In this study, the actual sample size was 127 subjects. Although a formal power analysis was not performed, sample sizes that meet or surpass minimum thresholds derived from population-based calculations such as those generated by tools like Raosoft are generally considered adequate for conducting valid descriptive and correlational analyses, particularly in educational and healthcare research contexts [22].

### Study setting

This study was conducted in the United Arab Emirates (UAE) at the Higher Colleges of Technology (HCT). HCT is a prominent educational institution in the UAE, with approximately 25,000 students enrolled across 16 campuses situated in different cities including Abu Dhabi, Dubai, Al Ain, Sharjah, Ras Al Khaimah, Fujairah, and Madinat Zayed. The student body at HCT is notably diverse, consisting of both male and female students from various nationalities and backgrounds. HCT encompasses seven academic divisions offering a total of 73 academic programs, reflecting the institution’s diversity and breadth of educational offerings. Since this study enrolled only nursing students with ICU clinical placement, the targeted population and sample was only recruited from campuses with nursing major which include Ras al-Khaimah, Sharjah, and Fujairah. The nursing programs across these three campuses only have females.

### Data collection procedure

The data collection process commenced in a period between 25 September 2024 and 29 October of 2024 after the approval from Research Ethics Integrity Committee (REIC) at Higher College of Technology. Validated questionnaires in former studies were administered via an online survey created using Microsoft Google Forms. Participants were provided with the survey link or QR code either through their HCT email or via personal invitation to access the survey. Additionally, the survey was shared via WhatsApp to reach out to students. Finally, students were provided with the study details (study aim, data collection procedure, data collection tool, risks and benefits). Those who agreed to participate signed informed consent.

#### Data collection tool.

The data collection instrument for this study comprises of the following questionnaires:

**Demographic data questionnaire:** This questionnaire was specifically designed for the purposes of this research study by the study researchers. It consists of nine questions that aim to gather general sociodemographic information from participants. The questions in the questionnaire inquire about the participant’s age, marital status, income, campus of the Higher Colleges of Technology (HCT), semester, number of critical care clinical courses, type of the last clinical placement, grade point average (GPA), and interest in nursing.

#### Perceived stress.

**Conceptual definition:** Perceived stress refers to an individual’s subjective assessment of the level of pressure, tension, and difficulty they experience in response to external stressors. This perception is influenced by personal beliefs, coping mechanisms, and social support networks. According to Cohen et al. (1983), perceived stress significantly impacts mental and physical health outcomes, underscoring its importance in evaluating overall well-being.

**Operational definition:** The perceived stress of nursing students was measured using the Perceived Stress Scale (PSS). This scale is a valid and reliable instrument developed by Sheu et al. (1997). This scale has 29 items that measure stress for six factors: stress from taking care of patients (8 items), stress from teachers and nursing staff (6 items), stress from tasks and workload (5 items), stress from peers and daily life (4 items), stress from lack of professional knowledge and skills (3 items), stress from environment/clinical setting (3 items). Each item has a 5-point Likert scale as follows: (0) never, (1) rarely, (2) sometimes, (3) frequently, and (4) always. The total scores could range between 0 and 116. A higher score indicated a higher degree of stress. The following criteria were used to determine the level of stress; low stress (0–1.33), moderate stress (1.34–2.66), and high stress (2.67–4.0). The original internal consistency of the PSS showed a Cronbach’s alpha between 0.86 and 0.89 (Sheu et al., 2002; Chan et al., 2009), with a content validity index of 0.94 (Chan et al., 2009). In our study, Cronbach’s alpha was 0.96 for the total PSS score, and ranged from 0.84 to 0.96 across the six domains, demonstrating excellent internal consistency. This instrument is available for use without any restriction.

#### Coping mechanisms.

**Conceptual definition:** Coping is the continually changing cognitive and behavioral efforts to manage certain external and/or internal demands that are deemed to be demanding or surpassing the person’s available resources (Sheu et al., 2002). Coping functions include controlling or changing the situation producing discomfort, as well as regulating the emotional reaction to the problem.

**Operational definition:** Coping strategies of nursing students was measured using the coping Behavior Inventory (CBI). This scale is a valid and reliable instrument developed by Sheu et al. (2002). This scale has 19 items that assess four major coping strategies: avoidance behavior (6 items), problem solving behavior (6 items), stay optimistic behavior (3 items), and transference behavior (3 items) (Appendix D). Each item has a 5-point Likert scale as follows: (0) never, (1) rarely, (2) sometimes, (3) frequently, and (4) always. Higher scores of each factor indicate more frequent use, and greater effectiveness of a certain type of coping behavior. The internal consistency of the CBI revealed Cronbach’s alpha of 0.76–0.80 (Chan et al., 2009; Sheu et al., 2002). In our study, Cronbach’s alpha was 0.94 for the total score of CBI, and it was 0.89, 0.92, 0.82, and 0.81 for the domains of avoidance, problem solving, stay optimistic, and transference, respectively. This instrument is available for use without any restriction.

### Ethical considerations

The research underwent ethical review by the HCT Research Ethics and Integrity Committee prior to initiation. Data collection only commenced after obtaining informed consent from participants. Participants’ identities remained anonymous, and no personal information such as names, emails, or phone numbers were requested. Participation in the study is entirely voluntary, and participants have the right to withdraw at any stage. The written consent form was obtained from all participants before their involvement.

The study offers no direct or indirect benefits and is not expected to cause any physical, emotional, or psychological harm to participants. Equal treatment was ensured for all participants, irrespective of age, race, nationality, or ethnicity. All collected information was kept strictly confidential and utilized exclusively for research purposes. Data were securely stored on password-protected devices accessible only to authorized researchers. Upon completion, the data was retained in the Higher Colleges of Technology research department database for a period of five years. The estimated time required to complete the study survey is 15–20 minutes.

### Data analysis

Data was analyzed using IBM SPSS version 27. The there was no missing data; however, the design and format of the Microsoft Form did not accept submissions with missing data. Descriptive statistics for categorical variables were presented using numbers and percentages, while continuous variables were summarized using the mean and Standard Deviation (SD) for quantitative data. Various statistical tests were employed. Independent t-test and one-way-ANOVA were used to compare averages of total score and domains of perceived stress scale (PSS) and coping behavior inventory (CBI). A stepwise multiple regression was used to determine the association between total scores and domains of perceived stress scale (PSS) and domains of coping behavior inventory (CBI), and demographics variables. Statistical significance will be determined using a threshold of P ≤ 0.05 for all analyses.

## Results

### Participant characteristics

Table 1 shows participants’ demographics and other academic and clinical-related characteristics. The response rate for this survey was 127 out of 180 (70.6%). The average age of participants was 22.13 (SD = 1.34). The majority were single (76.4%), had enough income (78%), and were from the Fujairah campus (48.8%). Most of the participants were interested in nursing (90.6%), placed in the critical care unit in their latest clinical rotation (70.1%), had three or more critical care courses (37.8%), and had a GPA between 2.1 and 3.0 (63.8%).

**Table 1:** Participants’ demographics and other academic and clinical characteristics (N = 127).

Variable		n (%) or Mean ± SD
Age		22.13 ± 1.34
Marital Status	Single	97 (76.4)
	Married	27 (21.3)
	Divorced	3 (2.4)
Income (self or family)	Enough	99 (78.0)
	Not enough	28 (22.0)
Campus	Sharjah	35 (27.6)
	Ras Al-Khaimah	30 (23.6)
	Fujairah	62 (48.8)
Semester level	Semester 7	59 (46.5)
	Semester 8	68 (53.5)
Number of critical care courses	One	34 (26.8)
	Two	45 (35.4)
	Three or more	48 (37.8)
Types of ward/units in the latest clinical placement	Critical Care Unit	89 (70.1)
	Non-Critical care Unit/Ward	38 (29.9)
GPA	0.0-1.0	2 (1.6)
	1.1-2.0	16 (12.6)
	2.1-3.0	81 (63.8)
	3.1-4.0	28 (22.0)
Are you interested in nursing?	Yes	115 (90.6)
	No	12 (9.4)

### Level of stressors perceived by students (questions 1 and 2)

Table 2 demonstrates the perceived level of stress and rank types of stressors among nursing students during their ICU placements. The average level for overall stress was 1.87 (SD = 0.80). The most common type of stressors among students was the perceived stress from assignments and workload (M = 2.12, SD = 0.91); the highest perceived stress was due to their worry about bad grades (M = 2.25, SD = 1.17), followed by inflexible clinical practice which affecting their social life (M = 2.18, SD = 1.25), and their felt that clinical requirements were exceeding their physical and emotional endurance (M = 2.15, SD = 1.119). The second most common type of stressor was related to stress from peers and quality of life (M = 1.98, SD = 1.03); the highest perceived stress in this domain was related to their felt of competition from peers in school and clinical practice (M = 2.09, SD = 1.24).

**Table 2:** Levels of perceived stressors by nursing students underwent ICU replacements (N = 127).

Stressor Factor/Items	Mean ± SD	Factor Ranking
**Overall PSS score**	**1.87 ± 0.80**	
**1. Stress from taking care of patients**	**1.73 ± 0.89**	**5**
1.Lack of experience and ability in providing nursing care and in making judgments	1.69 ± 1.15	
2.Do not know how to help patients with physio-psycho-social problems	1.76 ± 1.19	
3.Unable to reach one’s expectations	1.79 ± 1.13	
4.Unable to provide appropriate responses to doctors’, teachers’, and patients’ questions	1.64 ± 1.12	
5.Worry about not being trusted or accepted by patients or patients’ family	1.83 ± 1.06	
6.Unable to provide patients with good nursing care	1.57 ± 1.10	
7.Do not know how to communicate with patients	1.80 ± 1.25	
8.Experience difficulties in changing from the role of a student to that of a nurse	1.80 ± 1.18	
**2. Stress from teachers and nursing staff**	**1.86 ± 0.96**	**3**
9.Experience discrepancy between theory and practice	2.01 ± 1.20	
10.Do not know how to discuss patients’ illness with teachers, and medical and nursing personnel	1.98 ± 1.30	
11.Feel stressed that teacher’s instruction is different from one’s expectations	1.94 ± 1.22	
12.Medical personnel lack empathy and are not willing to help	1.81 ± 1.20	
13.Feel that teachers do not give fair evaluation on students	1.74 ± 1.25	
14.Lack of care and guidance from teachers	1.65 ± 1.24	
**3. Stress from assignments and workload**	**2.12 ± 0.91**	**1**
15.Worry about bad grades	2.25 ± 1.17	
16.Experience pressure from nature and quality of clinical practice	1.98 ± 1.12	
17.Feel that one’s performance does not meet teachers’ expectations	2.04 ± 1.14	
18.Feel that the requirements of clinical practice exceed one’s physical and emotional endurance	2.15 ± 1.19	
19.Feel that dull and inflexible clinical practice affects one’s family and social life	2.18 ± 1.25	
**4. Stress from peers and daily life**	**1.98 ± 1.03**	**2**
20.Experience competition from peers in school and clinical practice	2.09 ± 1.24	
21.Feel pressure from teachers who evaluate students’ performance by comparison	2.00 ± 1.19	
22.Feel that clinical practice affects one’s involvement in extracurricular activities	2.05 ± 1.22	
23.Cannot get along with other peers in the group	1.79 ± 1.23	
**5. Stress from lack of professional knowledge and skills**	**1.70 ± 1.08**	**6**
24.Unfamiliar with medical history and terms	1.69 ± 1.14	
25.Unfamiliar with professional nursing skills	1.66 ± 1.22	
26.Unfamiliar with patients’ diagnoses and treatments	1.76 ± 1.17	
**6. Stress from the environment**	**1.83 ± 1.02**	**4**
27.Feel stressed in the hospital environment where clinical practice takes place	1.94 ± 1.11	
28.Unfamiliar with the ward facilities	1.69 ± 1.25	
29.Feel stressed from the rapid change in patient’s condition	1.87 ± 1.20	

*Each item has a 5-point Likert scale as follows: (0) never, (1) rarely, (2) sometimes, (3) frequently, and (4) always. A higher score indicated a higher degree of stress.

**Fig 1:**
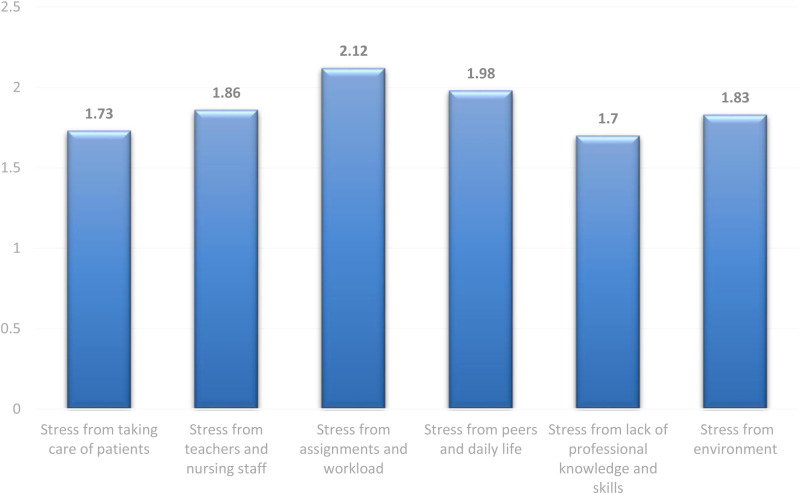
Level of perceived stressors among registered nurses underwent ICU placements (N = 127).

The third-ranked type of stressor among nursing students was due to the perceived stress from teacher and nursing staff (M = 1.86, Sd = 0.96), the highest perceived stress in this domain was related to their experience of discrepancies between theory and practice (M = 2.01, SD = 1.20), followed by their uncertainty of how to discuss patients illness with teachers and other healthcare professionals (M = 1.98, SD = 1.30). Fig 1 displays the scores of the six domains (factors) of perceived stress reported by the nursing students.

### Coping strategies frequently used by nursing students (question 3)

**Fig 2:**
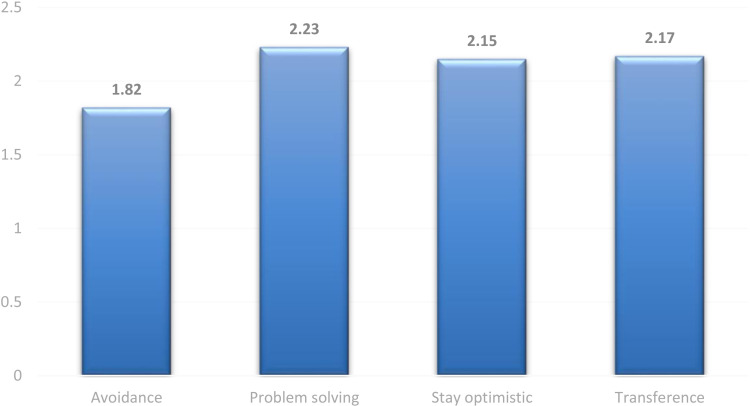
Coping mechanisms used by nursing students underwent ICU placements (N = 127).

Table 3 illustrates coping mechanisms used by nursing students during their ICU placements. Furthermore, Table 3 and Fig 2 rank different types of coping mechanisms. The most reported coping mechanism among students was the problem-solving mechanism (M = 2.23, SD = 0.95), followed by the transference mechanism (M = 2.17, SD = 1.00), and staying optimistic (M = 2.15, SD = 0.95). However, avoidance was the least reported coping mechanism (M = 1.82, SD = 0.95). Fig 2 displays the scores of the four coping mechanisms used by nursing students.

**Table 3:** Coping mechanisms used by nursing students underwent ICU placements (N = 127).

Stressor Factor/Items	Mean ± SD	Factor Ranking
**1. Avoidance**	**1.82 ± 0.95**	**4**
1.To avoid difficulties during clinical practice	1.83 ± 1.21	
2.To avoid teachers	1.72 ± 1.19	
3.To quarrel with others and lose temper	1.78 ± 1.15	
4.To expect miracles so one does not have to face difficulties	1.87 ± 1.17	
5.To expect others to solve the problem	1.91 ± 1.19	
6.To attribute to fate	1.81 ± 1.10	
**2. Problem solving**	**2.23 ± 0.95**	**1**
7.To adopt different strategies to solve problems	2.13 ± 1.11	
8.To set up objectives to solve problems	2.19 ± 1.09	
9.To make plans, list priorities, and solve stressful events	2.31 ± 1.14	
10.To find the meaning of stressful incidents	2.24 ± 1.15	
11.To employ past experience to solve problems	2.20 ± 1.22	
12.To have confidence in performing as well as senior schoolmates	2.29 ± 1.11	
**3. Stay optimistic**	**2.15 ± 0.95**	**3**
13.To keep an optimistic and positive attitude in dealing with everything in life	2.16 ± 1.13	
14.To see things objectively	2.25 ± 1.18	
15.To have confidence in overcoming difficulties	2.19 ± 1.17	
16.To cry, to feel moody, sad, and helpless	2.01 ± 1.25	
**4. Transference**	**2.17 ± 1.00**	**2**
17.To feast and take a long sleep	2.06 ± 1.15	
18.To save time for sleep and maintain good health to face stress	2.21 ± 1.15	
19.To relax via TV, movies, a shower, or physical exercises (ball playing, jogging)	2.24 ± 1.21	

*Each item has a 5-point Likert scale as follows: (0) never, (1) rarely, (2) sometimes, (3) frequently, and (4) always. Higher scores of each factor indicate more frequent use, and greater effectiveness of a certain type of coping behavior.

### Differences between stress level, and coping mechanisms across demographics (question 4)

Table 4 shows average total scores for perceived stress and coping mechanisms domains across the participants’ sociodemographic and other academic and clinical characteristics. Compared with students without enough income, those with enough income reported a higher perceived stress from the environment (1.95 ± 0.98 vs. 1.39 ± 1.05, t = 2.634, p = 0.010). Compared with those in semester 8, those in semester 7 reported a lower level of staying optimistic mechanism (1.97 ± 0.99 vs. 2.31 ± 0.90, t = -2.024, p = 0.045). It’s worth mentioning that only significant results were reported in this table. Furthermore, only significant results were reported in the table and the results for types of wards/units in the latest clinical placement and interest in nursing were not reported in this table since there were no significant findings in any domain of perceived stress or coping mechanisms.

**Table 4:** Independent t-test results for sociodemographic and other academic and clinical characteristics concerning stress and coping mechanisms (N = 127).

Outcome variables	Independent variables	Mean ± SD	df	T value	P value
	**Income (n: 99 enough, 28 not enough)**		125		
Stress from environment	Enough	1.95 ± 0.98		2.634	0.010^**^
	Not enough	1.39 ± 1.05			
	**Semester (n: 59 semester 7, 68 semester 8)**		125		
Stay optimistic	Semester 7	1.97 ± 0.99		-2.024	0.045^*^
	Semester 8	2.31 ± 0.90			

*Statistically significant at (α ≤ 0.05), **statistically significant at (α ≤ 0.01), ***statistically significant at (α ≤ 0.001).

The average scores for perceived stress and coping mechanisms domains were compared across the participants’ sociodemographic and other academic and clinical characteristics using one-way ANOVA. The average scores for stress from assignments and workload [F (2, 124) = 3.509, p = 0.033], stress from environment workload [F (2, 124) = 5.317, p = 0.006], overall score of stress [F (2, 124) = 3.107, p = 0.048], and transference coping mechanism [F (2, 124) = 5.484, p = 0.005] were found to be significantly different across the campus. Also, the average scores for stress from teachers and nursing staff were significantly different across students’ GPA [F (2, 124) = 2.752, p = 0.046]. Furthermore, only significant results were reported in this paragraph, and the results for marital status and number of critical care courses were not reported in this table since there were no significant findings in any domain of perceived stress or coping mechanisms.

### Association between perceived stress and coping behavior (question 5)

Table 5 shows factors associated with overall scores and domains of perceived stress. Avoidance and transference coping mechanisms were significantly associated with the overall score of PSS (R^2^ = 0.63). These two factors could explain 63% of the overall score of PSS among nursing students. One unit increment in the avoidance coping mechanisms increased the overall stress score by 0.55 units (B = 0.55; 95%CI: 0.45, 0.65, P < 0.001). Avoidance, transference, and income were significantly associated with stress from caring for patients (R^2 ^= 0.42). The avoidance mechanism was the only factor significantly associated with stress from teachers and nursing staff (R^2^ = 0.46). Avoidance, transference, and staying optimistic were significantly associated with stress from assignments and workload (R^2^ = 0.48). Both stress from peers and quality of life and stress from lack of professional knowledge and skills were significantly associated with avoidance and transference coping mechanisms (R^2^ = 0.22 and R^2^ = 0.46, respectively). Finally, avoidance, transference, problem-solving, and income were significantly associated with stress from the environment (R^2^ = 0.53). Avoidance and transference coping mechanisms were found to be significantly associated with overall scores of PSS and most of the domains of PSS.

**Table 5:** Factors associated with overall score and domains of perceived stress among nursing students underwent ICU placements (N = 127).

Items	B	SE	Beta	95% CI	P
**PSS overall score**					
Avoidance	0.55	0.05	0.65	0.45, 0.65	<0.001
Transference	0.20	0.05	0.24	0.07, 0.30	<0.001
Model summary: F = 103.91, P < 0.001, R^2 ^= 0.63, Adjusted R^2^ = 0.62.					
**Stress from taking care of patients**					
Avoidance	0.51	0.08	0.54	0.36, 0.67	<0.001
Income	0.36	0.15	0.17	0.07, 0.67	0.017
Transference	0.16	0.08	0.17	0.01, 0.31	0.046
Model summary: F = 29.18, P < 0.001, R^2 ^= 0.42, Adjusted R^2^ = 0.40.					
**Stress from teachers and nursing staff**					
Avoidance	0.69	0.07	0.68	0.56, 0.82	<0.001
Model summary: F = 105.76, P < 0.001, R^2 ^= 0.46, Adjusted R^2^ = 0.45.					
**Stress from assignments and workload**					
Stay optimistic	0.29	0.08	0.30	0.13, 0.46	0.001
Transference	0.26	0.08	0.29	0.11, 0.41	0.001
Avoidance	0.24	0.08	0.25	0.09, 0.39	0.002
Model summary: F = 37.36, P < 0.001, R^2 ^= 0.48, Adjusted R^2^ = 0.46.					
**Stress from peers and daily life**					
Avoidance	0.62	0.08	0.57	0.46, 0.77	<0.001
Transference	0.26	0.07	0.25	0.11, 0.40	0.001
Model summary: F = 65.38, P < 0.001, R^2^ = 0.22, Adjusted R^2^ = 0.21.					
**Stress from lack of professional knowledge and skills**					
Avoidance	0.63	0.09	0.55	0.46, 0.80	<0.001
Transference	0.23	0.08	0.22	0.07, 0.40	0.005
Model summary: F = 53.34, P < 0.001, R^2^ = 0.46, Adjusted R^2^ = 0.45.					
**Stress from the environment**					
Avoidance	0.64	0.08	0.59	0.49, 0.80	<0.001
Transference	0.36	0.09	0.35	0.19, 0.53	<0.001
Problem solving	-0.22	0.10	-0.21	-0.411, -0.04	0.021
Income	-0.35	0.15	-0.14	-0.65, -0.05	0.025
Model summary: F = 34.94, P < 0.001, R^2^ = 0.53, Adjusted R^2^ = 0.52.					

## Discussion

The current study aimed to assess perceived stress levels, identify coping mechanisms, and explore associations between stress, coping mechanisms, and demographic factors among nursing students during their Intensive Care Unit (ICU) clinical placement. This study’s findings on stressors and coping mechanisms among nursing students during ICU placements both align with and diverge from existing literature, offering a nuanced perspective on the stress landscape for students in high-intensity clinical environments. The overall perceived stress score of 1.87 ± 0.80 indicates a moderate level of stress, this is in consistency with previously conducted studies that have recorded similar levels of stress emanating from different clinical settings. For instance, Akhu-Zaheya, Shaban [23] found that nursing students experience moderate stress levels during clinical training. Ahmed and Mohammed [24] also reported similar findings in their study on Saudi nursing students. The ranking of stressors provides a clear hierarchy of the challenges that nursing students encounter, with stress from assignments and workload being the most significant factor (mean score of 2.12 ± 0.91).

This finding is supported by previous literature that highlights academic pressures among nursing students, underlining heavy workload and concerns about academic performance as common stressors that may also have negative consequences on students’ mental health and academic success [5,25]. The second most significant source of stress identified in the study is peer-related stress, with a mean score of 1.98 ± 1.03. Therefore, this finding confirms that nursing students have to go through a tough competitive atmosphere created by both peers and instructors, which may raise levels of inadequacy and anxiety [26]. The stressors linked to the transition from student to nurse, both in terms of an inability to meet expectations and a challenge in communicating effectively with patients, signal emotional and psychological demands during the clinical placements for nursing students [27,28].

Interestingly, the third in rank were stresses from teachers and nursing staff, with a mean score of 1.86 ± 0.96. It can thus be reasoned that the educational environment plays a leading part in the students’ stress-perceived incongruities between theory and practice being one of the leading causes. Al-Yateem, Griffiths [29] note that nursing students often feel unsupported by faculty, which may contribute to increased anxiety or decreased confidence in their clinical abilities. Hamadi, Zakari [12] also pointed out that minimal support from instructors’ results in undesirable effects on students’ learning processes. Moreover, the study reveals that stressors related to a lack of professional knowledge and skills, as well as environmental stressors, are also significant but rank lower in comparison to workload and peer-related stress. Similarly, the findings of Zhao, Lei [30] also pointed to knowledge gaps and environmental factors as sources of stress, they nonetheless suggested that, compared to academic performance demands and peer competition, these may not be as immediate or pressuring.

The overall mean score for coping mechanisms indicates a diverse range of strategies utilized by students, with problem-solving emerging as the most favored approach (mean score of 2.23 ± 0.95). This finding aligns with previous studies, which have found effective coping strategies, particularly the problem-focused approaches, as important in minimizing the level of stress experienced, especially in the high-pressure nursing environment [30]. Different strategies employed to solve problems and set objectives require that nursing students should not only maintain their existing level of stressors but also be resilient and adaptable during the clinical practice process according to Hamadi, Zakari [12]. The second most frequently used coping strategy was the transference coping strategy, with a mean score of 2.17 ± 1.00; it covers strategies such as spending leisure time, keeping good health, and finding time to relax. This assertion of transference as a coping mechanism is reiterated by a finding from Chin, Ching [31], who indicated that nursing students most often utilize relaxation techniques to minimize stress during clinical practice. This means that although problem-solving remains key, the students also recognize the need for self-care in stress relief so that their whole being can be maintained.

The third-rated coping behavior was staying optimistic, with a mean score of 2.15 ± 0.95, which denotes that one should not lose hope in the face of challenges. This finding is supported by previously established literature [32] that optimism acts as a protective factor against the occurrence of stress and strengthens one’s coping behavior. Optimism and building confidence in themselves help students be at ease during demanding clinical practices. Avoidance coping ranked fourth with a mean score of 1.82 ± 0.95. Gibbons, Dempster [33] highlighted that reliance on avoidance coping is linked to lower satisfaction and higher levels of stress among nursing students and nothing can be defined as the way of attaining long-term success in the nursing profession. Additionally, Al‐Zayyat and Al‐Gamal [19] found that the usage of avoidance strategies might be associated with an increase in subjective levels of stress.

Regarding differences in perceived stress levels and coping mechanism. Students with sufficient income reported higher levels of perceived stress from the environment (1.95 ± 0.98) compared to those without sufficient income (1.39 ± 1.05), with a statistically significant difference (t = 2.634, p = 0.010). This finding suggests that financial stability does not correlate with lower levels among nursing students, a finding that was opposite of the expected.. For instance, Varghese et al. (2022) reported that among nursing students, there was a frequent presence of moderate academic stress, which was possibly further exacerbated by financial pressures and expectations concerning their performance in education. One possible explanation is that students with sufficient income may have higher expectations for their clinical environment and resources, leading to increased sensitivity to environmental stressors.

Furthermore, the results indicate that students in semester 7 reported a lower level of the coping mechanism of staying optimistic (1.97 ± 0.99) compared to those in semester 8 (2.31 ± 0.90), with a significant difference (t = -2.024, p = 0.045). This result may reflect the cumulative stress students undergo during their study. The rather advanced stages of study might be associated with higher academic and clinical demands that have a harmful effect on the students’ optimistic orientation. Ahmed, Abdulla [34] found increased levels of stress among nursing students during their clinical placements, which may lower their coping strategies, such as optimism.

Concerning the differences in perceived stress levels and coping mechanisms among nursing students based on various academic and sociodemographic characteristics. Assignment and workload-related stress varied significantly across campuses: F (2, 124) = 3.509, p = 0.033. This indicates that the academic environment may act as a modifying variable regarding the level of stress perceived by students. This finding supports prior research by Yildirim‐Hamurcu and Terzioglu [35], who found that nursing students always identified assignments and workload as two major contributors of stress during clinical practice. More specifically, this may be partly explained by campus variation due to academic expectations, faculty support, or resources available. Moreover, stress from the environment was found to be significantly different across campuses (F (2, 124) = 5.317, p = 0.006).

This suggests that environmental factors, such as the physical setting of the campus or the availability of clinical placements, may play a crucial role in shaping students’ stress experiences. Previous research has mentioned the characteristics of the clinical setting as contributing factors in the level of nursing students’ stress, in which the presence of a supportive and adequately resourced environment may reduce stress [35]. The overall score of stress also showed significant differences (F (2, 124) = 3.107, p = 0.048), reinforcing the notion that various factors contribute to the cumulative stress experienced by nursing students. This is consistent with findings from Shahin [36], that various stressors such as academic workload, environmental condition where they learned, collectively add to their perceived scores of stress. The transference coping mechanism also varied significantly across campuses: F (2, 124) = 5.484, p = 0.005. This may imply that students use different coping strategies according to campus environment. Transference coping involves leisure activities one induces and practice of self-care, which is very fundamental in managing stress effectively. The literature denotes the role of coping strategies in lessening stress, for instance, finding that effective coping mechanisms ensure better mental health outcomes among nursing students [37]. Furthermore, the stress from teachers and nursing staff differed significantly regarding the GPA of students, F (3, 123) = 2.752, p = 0.046. With this result, lower or higher academic performance might indicate different levels of stress as related to faculty interactions. Research has indicated that students with lower GPAs have many times been found to exhibit high levels of stress emanating from academic pressures and expectations of faculty members [38]. In addition, Alsaqri [39] reported that nursing students identified clinical instructors as a significant source of stress, especially when guidance was perceived as unsupportive. Similarly, Alzayyat and Al‐Gamal [40] highlighted that faculty-student dynamics were among the most frequently reported stressors in clinical education.

Associations between perceived stress and various coping mechanisms among nursing students. Significantly, avoidance and transference coping mechanisms contributed to the overall score of the PSS, explaining 63% of variance in stress levels (R2 = 0.63). This suggests that students who practice avoidance mechanisms (those denying or procrastinating in the face of stressors) show higher levels of overall stress. It is supported by previous research [41]. Furthermore, the analysis indicated that with each one-unit increase in avoidance coping mechanisms, the overall score for stress was raised by 0.55 units. This finding concurs with that of Sun, Xu [42] who found work that reliance on the avoidance coping mechanism among nursing students leads to higher levels of perceived stress. In addition, the study found that avoidance and transference coping mechanisms were significantly associated with stress from caring for patients. This means that students experiencing much care-related stress may struggle more emotionally while caring for patients, using these coping strategies. The findings are supported by research from Kim [43], which indicated that the nursing students experience increased levels of stress toward caregiving activities, especially when utilizing maladaptive coping styles. The avoidance mechanism, however, was the only factor that significantly related to stress from teachers and nursing staff. This clearly indicates that the students might be overwhelmed by the demands expected of them and use the avoidance mechanism for coping, hence may not perform well academically and professionally. This is further supported by Yildirim‐Hamurcu and Terzioglu [35] who found that stress arising from relationships with the faculty results in increased use of strategies of avoidance among students of nursing.

The results also indicated that avoidance, transference, and staying optimistic were significantly associated with stress from assignments and workload. This highlights the multifaceted nature of stressors in nursing education, whereby academic demands may predispose nursing students to various coping responses. Research by Malik and Javed [44] supports this notion, it points out that among the major sources of stress among nursing students is academic pressure, often leading to the use of both adaptive and maladaptive coping strategies. The study also found that avoidance and transference coping mechanisms were significantly associated with stress from peers and quality of life, as well as stress from a lack of professional knowledge and skills (R² = 0.22 and R² = 0.46, respectively). It suggests that students with problems with peer relationships may exhibit more avoidance and transfer coping. The literature has pointed to the importance of creating a positive sense of peer relationships, due to the significant effect of the aforementioned factors on the levels of stress and well-being [44].

### Implications

The main implications of the study are as follows. In institutional level interventions, the main stressors, namely, workload, peer relationships, and relations with staff, should be addressed to reduce stress among nursing students during ICU placements. The promotion of effective coping strategies alone, especially problem-solving strategies, would help students cope with stress in a more constructive manner. This indicates a specific need for support systems, such as financial aid and academic counseling services, regarding stress levels associated with demographic factors like financial stability and GPA. In addition, the training program should cover methods for managing stress that would provide students with resilience in highly pressurized clinical environments and thus improve their well-being. Such steps may simply result in improved learning and mental health for nursing students. These findings can inform nursing education reforms by integrating structured stress management training and peer support systems within the curriculum. Curricular adjustments that promote active coping strategies and reduce unnecessary academic and clinical workload could directly benefit student outcomes. Furthermore, implementing institutional policies that foster a more inclusive and supportive learning environment particularly for students from diverse socioeconomic backgrounds can play a vital role in reducing stress related disparities.

### Limitations

The study design is cross sectional, and hence, there are limited possibilities of establishing causal relationships among stress and coping mechanisms and demographic variables; there is the bias associated with subjective perceptions or data based on social desirability since the data are based on self-report measures. Additionally, the use of a convenience sampling strategy should be acknowledged as a limitation, as it may introduce selection bias and affect the representativeness of the sample. The sample was confined to nursing students attending clinical placements in the ICU from a single setting; hence, there is lesser generalizability to populations and other clinical settings. The last factor, but not least important, is that no possible confounding factors were controlled in the study: already existing mental health conditions could produce the same result or differences in clinical supervision.

## Conclusion

This study highlights the moderate stress levels nursing students experience during ICU placements, with workload and pressures from peers as the most intense stressors. Problem-focused adaptive strategies reduced the level of stress while reliance on maladaptive avoidance strategies greatly increased the level of stress. Additionally, avoidance and transference coping were strong predictors of stress further underline the importance of effective coping skills in nursing education. Instructors can promote students’ wellbeing, enhance their clinical performance, and more adequately prepare them for the demands of critical care nursing by including stress management and resilience building into clinical training.

## Supporting information

S1 Data Revised data PLOS ICU. (XLSX)
